# Metal Recovery from Lunar Regolith via Deep Eutectic Solvent Electrolysis for In Situ Resource Utilization

**DOI:** 10.3390/ma19143120

**Published:** 2026-07-21

**Authors:** Vesna S. Cvetković, Nataša M. Petrović, Ksenija Milicevic Neumann, Bernd Friedrich, Jovan N. Jovićević

**Affiliations:** 1 Department of Electrochemistry, Institute of Chemistry, Technology and Metallurgy, University of Belgrade, Njegoševa 12, 11000 Belgrade, Serbia; vukicevic@ihtm.bg.ac.rs (N.M.P.); jovicevic@ihtm.bg.ac.rs (J.N.J.); 2 CEOS UG, Ottobrunner Str. 33, 81737 Munich, Germany; ksenijam87@gmail.com; 3 IME Process Metallurgy and Metal Recycling, RWTH Aachen University, Intzestrasse 3, 52056 Aachen, Germany; bfriedrich@metallurgie.rwth-aachen.de

**Keywords:** lunar regolith simulant, regolith solubility in DES, electrolysis, metal extraction, silicon, ethaline

## Abstract

**Highlights:**

- Lunar regolith was dissolved in the deep eutectic solvents, reline or ethaline.
- Reline demonstrated better dissolution efficiency for regolith compared to ethaline.
- A deposit predominantly composed of silicon was successfully extracted by precise control of deposition potential.
- Aluminum extraction required a higher deposition overpotential.

**Abstract:**

Sustaining human presence on the Moon depends on access to strategic metals, which can be achieved by directly utilizing extraterrestrial resources through in situ resource utilization (ISRU). This study presents novel insights and preliminary results into a previously unexplored strategy for metals extraction from the lunar regolith simulant Lunar Mare Soil (LMS-1) using deep eutectic solvents (DESs). Based on inductively coupled plasma–optical emission spectrometry (ICP-OES) measurements, the solubility of major oxide components of the regolith, SiO_2_, Al_2_O_3_, TiO_2_, Cr_2_O_3_, MgO and FeO_T,_ was investigated in ethaline (choline chloride:ethylene glycol, ChCl:EG) as well as reline (ChCl:Urea). Although both DESs enabled oxide dissolution, reline exhibited significantly higher dissolution efficiency, due to the additional hydrogen-bond donor sites, NH and CO groups from urea, as well as high chloride activity in the reline. Cyclic voltammetry (CV) and square wave voltammetry (SWV) revealed that dissolved metal species in the reline–regolith system undergo complex multivalent redox transitions. The equilibrium potentials of the metals were determined and correlated with the order in which the metals should be electrodeposited on the cathode from an electrolyte containing dissolved lunar regolith. Based on the data from electrochemical measurements, parameters for electrolysis were selected. At less negative overpotentials, the deposit consisted mainly of Si, while Al, Cr, and Fe, along with Si, were electrodeposited at more negative potentials. The results highlight the importance of considering the selective electrochemical extraction of metals from DESs using lunar regolith as the source.

## 1. Introduction

The demand for strategic materials required to establish a sustainable human presence on the Moon depends on the economically efficient use of in situ resources [1,2,3,4]. The most efficient approach for producing oxygen and metals is to conduct extraterrestrial operations beyond Earth, using resources directly available on the Moon [5]. The material that covers the surface of the Moon is regolith and corresponds to 4–5 m depths in Mare or 10–15 m depths in Highland regions [6]. Its widespread occurrence on the Moon makes it a highly promising raw material for such applications used to support construction, manufacturing, or radiation shielding [7]. The main focus is on extracting oxygen and valuable metals from the regolith which contains significant amounts of metal oxide compounds: 40.6 to 48.1 wt% SiO_2_, 6.3–28.0 wt% Al_2_O_3_, 4.7–24.5 wt% FeO_T_, and up to 8 wt% TiO_2_ [1,8,9]. However, despite its abundance as a valuable source of metals and oxygen, the feasibility of producing oxygen and metals via ISRU is challenging due to the complex, energy-consuming, and environmentally hazardous processes involved [6,10].

Currently, molten salt electrolysis (MSE) is the most commonly used approach to obtain oxygen and high-purity metals from terrestrial materials [3,5,10,11]. The Magma process, which uses direct molten salt electrolysis of lunar regolith, has been well studied at operating temperatures between 1573 and 1848 K [9,12,13]. However, the main limitations of the Magma process include reduced current efficiency when iron is present in the melt due to the oxidation of Fe^2+^ to Fe^3+^ [12], as well as high operating temperatures and difficulties in selecting anode materials [9,13]. In addition to the Magma process, the solid-state electrochemical reduction of metal oxides to metals in molten CaCl_2_ at temperatures around 1173 K, using the Cambridge process-FFC (Fray, Farthing, Chen), has also been undertaken [14]. Meurisse et al. demonstrated that the reaction efficiency is influenced not only by the operating temperature but also by the CaCl_2_ solubility in the molten salt [2]. Molten salt electrolysis using fluoride electrolytes for extracting metals from lunar regolith was also initiated as fluoride melts have good solubility for oxide raw materials [1,15]. The results disclosed that the solubility of Al_2_O_3_ was 2.8 wt%, while SiO_2_ exhibited 17.4 wt% solubility [1]. Another approach, aluminothermic reduction combined with electrolysis using molten cryolite, has also been investigated as a method for recovering valuable metals from lunar material [15]. The product obtained from the aluminothermic reduction of dissolved Minnesota Lunar Simulant-1 (MLS-1) and metallic aluminum at 1253 K in cryolite contained Al, Si, Al_5_FeSi, while the cathodic product was primarily an Al phase [15]. Currently, studies of extracting metals from lunar regolith via fluoride-based molten salt electrolysis are focused on using an inert anode [16].

Despite the feasibility of extracting metals from lunar regolith via high-temperature molten salt electrolysis, a new approach has been directed toward ionic liquid media, as they show potential for dissolving metal oxides present in the regolith [11,17,18]. DESs are of particular interest for lunar material processing because of their negligible vapor pressure and stability as liquids under the extreme conditions found on the Moon [11]. Compared to MSE, a several practical advantages make DESs convenient for ISRU processes of lunar regolith. The key advantages are the availability of components, lower thermal energy requirements, reduced operational risks for human crews, and the ability to lower the economic investments [11,17,18,19]. Rohde et al. investigated metal extraction from lunar regolith EAC-1 using 1-ethyl-3-methylimidazolium hydrogen sulfate [20]. Oxygen was successfully extracted by the ISRU method, although no metallic deposit was observed on the carbon working electrode after potentiostatic electrolysis at temperatures below 373 K [20]. Riviera et al. studied the dissolution of metals from two chondrites (H3, H5) and one IAB-MG meteorite in DES. Using oxidants such as iodine or FeCl_3_ in a choline chloride and ethylene glycol, Fe–Ni-rich phases were effectively leached, while other mineral phases remained largely unaffected [11]. These studies demonstrate the viability of using DES for sustainable metal recovery from lunar soil in extreme environments [11].

To the best of our knowledge, this study is the first to report the dissolution of lunar regolith simulant in choline chloride-based DESs, followed by the electrochemical recovery of metals from the resulting electrolyte. The novelty of this approach lies in integrating the dissolution of oxide constituents of lunar regolith with electrochemical metal recovery, thereby establishing a foundation for the selective extraction of valuable metals from DES-based regolith systems. Previous ionic liquid-based approaches either did not yield metallic deposits [20], or were confined to leaching without electrodeposition [11], leaving the electrochemical recovery of metals from DES–regolith systems entirely unexplored.

The aim of the study is to investigate the cathodic processes in electrolysis after lunar regolith dissolution in reline. To address this, regolith was dissolved in ethaline or reline. The equilibrium potentials of metals in the solvent containing dissolved regolith were determined and the electrochemical behavior of dissolved metal species in the reline–regolith system was investigated. The feasibility of obtaining metals on the cathode was studied using potentiostatic electrolysis.

## 2. Materials and Methods

Choline chloride (ChCl, ≥98%) and ethylene glycol (EG, 99.8%) were obtained from Sigma Aldrich (Burlington, MA, USA) and used as received. Urea (CH_4_N_2_O, p.a., Carlo Erba, Milan, Italy) was dried for 24 h (at 375 K) before electrolyte preparation. Ethaline was synthesized by mixing ChCl and EG, while reline was prepared from ChCl and urea, both in a 1:2 molar ratio, under magnetic stirring at 323 K until a homogeneous, colorless solution was obtained, as previously described in the details [21]. The method for obtaining electrolytes is based on dissolving LMS-1 (1 g LMS-1) in the freshly prepared (50 mL) ethaline (ChCl:EG) or reline (ChCl:Urea). The electrolytes then were stirred at 300 rpm using a magnetic stirrer for 10 h at 343 K under a continuous flow of argon. LMS-1 is a lunar regolith simulant prepared using information from the returned Apollo lunar soil samples to represent mare regions, which are the darker areas on the Moon’s surface. For researchers, coming into possession of genuine lunar regolith remains challenging, so researchers use lunar regolith simulant, prepared based on the composition and properties of the returned Apollo lunar soil samples. Due to the lack of actual lunar regolith, researchers typically utilize a lunar regolith simulant produced by NASA’s Johnson Space Center or Space Resource Technology. In this study, LMS-1 lunar regolith simulant, supplied by Space Resource Technology (Oviedo, FL, USA), was used. The supplier also provided a material specification detailing the simulant’s composition. Sometimes, the amount of P_4_O_10_ and the alkali minerals Na_2_O and K_2_O in the samples is higher than in lunar samples. This is a common problem for most lunar simulants, except NAO-1 [20]. Nevertheless, LMS-1 is very well suited to research metal extraction since predominantly major oxide fractions like, e.g., Si, Al, Ti, and iron oxide, are of particular interest for these investigations. To better match lunar conditions, the Apollo data were adjusted by excluding particles larger than 1 mm, resulting in a particle size range of 0–1 mm and an average particle size of 63 μm.

A laboratory designed a three-electrode airtight Pyrex glass electrochemical cell with a sealed lid containing ports for electrodes, a thermocouple (located in a glass tube), and gas supply connections was used [21]. Electrochemical measurements and electrolysis experiments were performed in the supernatant layer using gold as the working electrode (WE). For electrochemical measurements, a 2 mm diameter gold wire (Au, 99.999%, Alfa Aesar, Haverhill, MA, USA) was used, whereas a Au plate (0.5 cm^2^) served as the WE for electrodeposition experiments. A platinum plate (Pt, with an immersed surface area of 4.5 cm^2^) was used as the counter electrode. A platinum wire (Pt, 2 mm diameter, 99.99%, Sigma-Aldrich, Burlington, MA, USA), served as the quasi-reference electrode. The potentials of the Pt quasi-reference electrode were calibrated to the ferrocene/ferrocenium redox couple (F_C_/F_C_^+^), as described in [22]. During the open-circuit potential (OCP) measurements conducted in this study, the measured potential remained stable, varying by only ±5 mV over 30 min.

Before experiments, the electrodes were prepared according to previously established procedures for Au [23] and for Pt [24]. Dissolved oxygen in the system was removed by purging argon gas, and an argon flow (1.0 L/min) was kept throughout all experiments. As for the equilibrium potential measurements, the electrode was prepared by welding a copper wire to the corresponding metal plate to provide electrical contact, after which the assembly was sealed in resin within a glass tube. Pt wire was used as a quasi-reference electrode for these measurements, and the same Pt electrode was employed in the subsequent electrochemical experiments. A computer-controlled potentiostat (Gamry Interface 1010E model (Gamry Instruments Inc., Warminster, PA, USA)), operated via Gamry Echem Analysis-based software (Version 7.8.4.8183), enabled precise current/voltage control and data acquisition. Electrochemical techniques in this study include cyclic voltammetry (CV) and square wave voltammetry (SWV) carried out in a supernatant layer of reline electrolyte containing ions added by dissolution of lunar regolith. CV experiments were performed within a particular potential window for both electrolytes. The potential scan started at the initial potential (E_I_), typically 50 mV more negative than the open-circuit potential of Au in the chosen DES (measured versus the Pt reference electrode), swept to the cathodic potential limit (E_F_), and then returned to E_I_. For SWV, the measurements were repeated at various frequencies (50 and 60 Hz) to assess the optimal detection of analyte signals. All electrochemical experiments conducted in DESs and ICP-OES analyses were performed with at least three independent measurements (n ≥ 3), and the reported values represent the average of these measurements.

The metal content in both reline and ethaline supernatant layers after regolith dissolution and in the samples after electrodeposition was determined using ICP-OES analysis performed by an accredited laboratory in accordance with ISO standards using Spectro Spectrogreen TL-FMT 46 (SPECTRO Analytical Instruments, AMETEK Group, Berwyn, PA, USA). For accurate measurements, the undissolved LMS-1 was removed from the electrolyte by vacuum filtration prior to analysis. Electrodeposition experiments were performed under potentiostatic conditions. After deposition, the WE was removed from the electrochemical cell while maintaining the applied potential to preserve the deposited material. The WE was then thoroughly rinsed with absolute ethanol and dried. The quantities of metals in the solid cathodic deposit were determined after dissolving the deposit in a mixture of acids (aqua regia), followed by ICP-OES analysis. The morphology of the deposits was examined using scanning electron microscopy (SEM; TESCAN VEGA 3, Brno, Czech Republic). Energy-dispersive spectroscopy (EDS; Oxford INCA 3.2, High Wycombe, UK) was used for determining the elemental composition of the cathodic product. Philips PW 1050 powder diffractometer (XRD, Philips, Delft, the Netherlands) with Ni-filtered CuKα radiation (wavelength = 1.54178 Å) was used to determine the phase in the deposit.

## 3. Results

### 3.1. Solubility of the Oxides in Regolith

The solubility of oxides originated from lunar regolith, in the ethaline or reline electrolytes, was evaluated by measuring the concentrations of individual metal elements in the supernatant layer of the electrolytes using ICP-OES analysis, at 343 K [11,21]. After determining the metal concentrations in ethaline or reline, the solubilities of oxides, TiO_2_, Al_2_O_3_ FeO_T_ (calculated as FeO), SiO_2_, Cr_2_O_3_, were calculated, and the results are presented in Table 1. The calculation was performed according to the procedure described [1], by converting the concentrations of the individual metals in the supernatant layer to the corresponding masses of their metal oxides and comparing these values with the corresponding metal oxide mass fractions in the regolith. Various iron oxides FeO, Fe_2_O_3_, and Fe_3_O_4_ are present in the lunar regolith, but their relative amounts remain uncertain. FeO was used as an approximation to represent the total dissolved iron from all iron oxide species [1].

According to ICP-OES analysis of the supernatant layer of ethaline, the solubility of Al_2_O_3_ 0.3 wt%, (±2.66%) was lower compared to other oxides. SiO_2_ had a solubility of 0.5 wt% (±2.65%), Cr_2_O_3_ showed a solubility of 8.35 wt% (±2.44%), and FeO_T_ and MgO had solubilities of 1.63 wt% (±1.36%) and 2.89 wt% (±1.0%), respectively. The solubility of TiO_2_ was not determined because the analytical signal values obtained made the results unreliable. Since the electrochemical cell was constructed from Pyrex glass, blank experiments were conducted with pure ethaline and reline electrolytes. ICP-OES analysis detected no silicon in these blank electrolytes, indicating that Si dissolution from the glass cell did not occur under the experimental conditions. The Si concentration measured in the samples collected from the supernatant layer of the chosen electrolyte can therefore be attributed solely to the SiO_2_ dissolved from the regolith. Although the solubility of the examined oxides from the regolith was relatively low, the data recorded indicated that regolith dissolution is feasible under the specified parameters. Contrary to ethaline, the solubility of the oxides in reline was significantly higher. In reline, Al_2_O_3_ showed a solubility of 0.4 (± 2.66%) wt%, while SiO_2_ reached a solubility of 0.98 (±2.65%) wt%. Among the other components, in reline, Cr_2_O_3_ exhibited the highest solubility of 14.6 (±2.44%) wt%, followed by FeO_T_ with 2.27 (±1.36%) wt% and 4.1 (±1.0%) wt% for MgO. Since the obtained values for TiO_2_ solubility were considered unreliable, the solubility was also not determined. It appears that the solubility of each metal oxide is significantly reduced due to the joint solubility effect that occurs when multiple oxides are present simultaneously, as in regolith. This phenomenon is similar to that observed in the molten fluoride mixtures, suggesting that the species distribution is influenced by the composition and cation considered. The presence of free fluorine ions enables the formation of anionic complexes, as well as the formation of long chains by connecting different complexes via fluoride ions [25]. In contrast to the fluoride melts, in choline chloride-based electrolytes, chloride ligands predominantly form coordinated complexes with metal ions [26,27]. Although this interaction decreases the concentration of free metal ions and would generally be expected to increase solubility, metals in ethaline may also form complexes with ethylene glycol molecules, [M^x+^(EG)_y_]^x+^, rather than with chloride anions [28]. Such interactions likely induce a rearrangement of the solvent structure and contribute to an increase in viscosity, thereby reducing the individual solubility [27,28]. The slightly different coordination form of metal ions with reline components is expected due to the high chloride activity in the reline [29,30]. Urea molecules provide additional hydrogen-bond donor sites (NH, CO groups), and very strong hydrogen bond interactions are present between all reline components [26]. This effect may explain the experimentally observed increase in oxide dissolution from the regolith in the choline chloride-based electrolyte containing urea compared to ethylene glycol. From one point of view, the relatively low dissolution efficiency may be considered a limitation for practical ISRU applications. However, the electrolyte components are inexpensive and can potentially be produced from waste-derived materials. Moreover, the electrolyte preparation is simple, requires low energy consumption and does not involve hazardous conditions for the human crew. The advantages of the abundance of lunar regolith and the possibility of producing metals in situ may be enough to overcome the limitation associated with the low dissolution efficiency. Evidently, the solubility of oxides in reline was higher, so we chose to conduct further experiments in reline.

### 3.2. Electrochemical Behavior

The electrochemical analysis of the electrolyte containing various metallic ions after lunar regolith dissolution is expected to produce a complex picture, making it difficult to clearly identify the individual reactions involved. To address this, it was first necessary to determine the individual reversible (equilibrium) potentials of the investigated metals in a reline electrolyte containing dissolved regolith. These values provide a basis for formulating hypotheses about the sequence of onset electrochemical reduction potentials for the metals and for understanding how they influence the overall electrochemical response of the system. The measurements were performed using the same platinum reference electrode as in all electrochemical experiments, and the working electrode was a laboratory-made electrode of the selected metal. The most negative potential was observed for Mg, E_Mg_ = −1.280 V vs. Pt, followed by the equilibrium potential of Al, E_Al_ = −0.580 V vs. Pt. Approximately 700 mV difference between the equilibrium potentials of these two metals suggests that the onset deposition potential of Al and Mg can be clearly distinguished. This indicates that adjusting the electrodeposition potential could effectively prevent the deposition of Mg while enabling the deposition of Al. The equilibrium potential of Si was more positive E_Si_ = −0.250 V vs. Pt than the equilibrium potential of Al, indicating that although the potential difference is smaller, it is still possible to distinguish the onset deposition potentials of Al and Si. At more positive potentials, the equilibrium potentials of Cr, E_Cr_ = −0.144 V vs. Pt and Fe, E_Fe_ = −0.110 V vs. Pt were measured, indicating that their electrochemical reduction reactions occur at very-close potentials or in parallel. The equilibrium potential of Mn was not determined. For the remaining dissolved metals, their deposition potentials are likely outside the electrochemical stability window of the electrolyte. This finding is particularly significant because the experimentally measured equilibrium potentials in the reline system follow the same order as the standard electrode potentials of the corresponding metals referenced to the normal hydrogen electrode (NHE) [31], despite the differences in their absolute values. Accordingly, the theoretical sequence of metal deposition on the cathode via electrolysis from dissolved lunar regolith in reline is expected to be as follows: Fe, Cr, Si, Al, and Mg.

**Figure 1:**
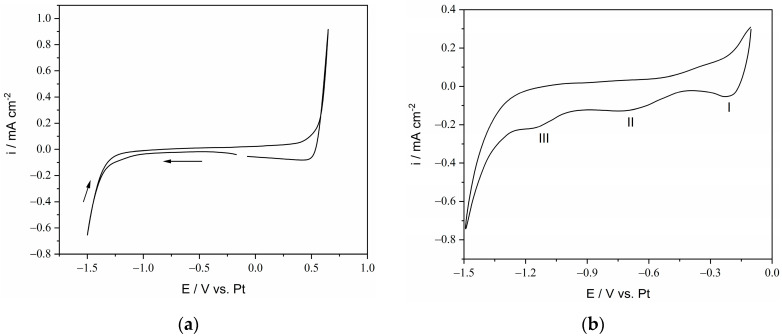
Voltammetric responses of: (**a**) pure reline and (**b**) reline + regolith electrolyte on Au working electrode; potential scans end at cathodic potential of −1.500 V vs. Pt; scan rates 20 mV s^−1^; temperature 343 K.

To clarify the electrochemical behavior of the system, which is reline containing dissolved regolith at 343 K, and identify possible redox reactions related to the metal electroreduction from the electrolyte, CV was performed. The voltammetric response on a gold wire WE in the pure reline with a scan rate of 20 mV s^−1^, is shown in Figure 1a. The CV was recorded over a potential range of 0.600 V to −1.500 V vs. Pt. As shown on the voltammogram, the absence of cathodic and anodic waves during the scan confirms that no electrochemical reactions occurred in pure reline. The potentials at which the current density begins to increase are attributed to the anodic or cathodic decomposition of reline components. After dissolving regolith in the reline, the CV recorded reveals three additional reduction peaks within the potential range 0.000 V and −1.500 V vs. Pt, as shown in Figure 1b. In the system, regolith dissolved in reline, Au cathode, Pt counter and Pt reference electrode, the CV response at an electrode potential lower than −0.100 V and a scan rate of 20 mV s^−1^ does not clearly reflect individual redox processes; the well-defined shape of the cathodic waves was not observed, as seen in Figure 1b. Within the applied electrode potentials, the cathodic peak (I) is observed at around −0.200 V vs. Pt, and without clear separation, continues the broad second peak (II) spanning from −0.440 V to −0.820 V vs. Pt. It appears that the redox transitions of Fe and Cr ions in the electrolyte, probably represented by cathodic current wave I, occur at potentials very close to those of the redox transitions of Si ions (probably associated with cathodic current wave II) in the electrolyte, indicating that the reduction processes likely occur simultaneously. The potential associated with the third cathodic peak (III), appearing around −1.150 V vs. Pt, probably reflects the further redox transitions of Fe, Cr, and Si, along with the electroreduction of Al, which has a more negative reduction potential than those metals in the electrolyte. The reduction of Mg(II) is also possible, but it is unlikely that the redox potential of Mg(II) is reached at −1.150 V, since Mg deposition potential can be shifted toward more negative values by approximately 0.400 V in DES, as reported in [30]. If metal electrodeposition proceeds through multiple reduction steps, several electrochemical processes occur within a relatively small potential interval, resulting in broad, less distinct cathodic current waves, making the identification of clear deposition onset potentials very difficult. The equilibrium potentials measured in this study for the reduction reactions of Fe^x+^/Fe, Cr^3+^/Cr, and Si^4+^/Si are very close in value; it is most probably that the next reduction process commences while the previous process is proceeding. As a result, the general characteristics of the voltammetry responses in this specific case cannot reflect all the redox transitions [1,32]. For this reason, at the current stage of the investigation, it is not possible to unambiguously assign the individual contributions of each peak observed in the CV voltammograms to each redox reaction.

When the potential scan direction was changed, the CV scan showed no anodic oxidation peaks related to the dissolution of the metals electrodeposited from the reline containing dissolved lunar regolith. The possible explanation for this behavior interprets that the electrochemical oxidation of deposited metals is a highly irreversible process, and the dissolution process is inhibited within the electrochemical window of this electrolyte [22]. This was also noted by Pulletikurthi et al. in their study of the electrochemical deposition of Si from SiCl_4_ in various pyrrolidinium-based ILs at both room temperature and 373 K [22]. For this phenomenon, the authors concluded that the oxidation of the working electrode, accompanied by chlorine formation from SiCl_4_, is the main reason for the absence of anodic peaks on the CV [22]. These observations imply that choline chloride-based DESs may exhibit electrochemical behavior comparable to pyrrolidinium-based ILs.

**Figure 2:**
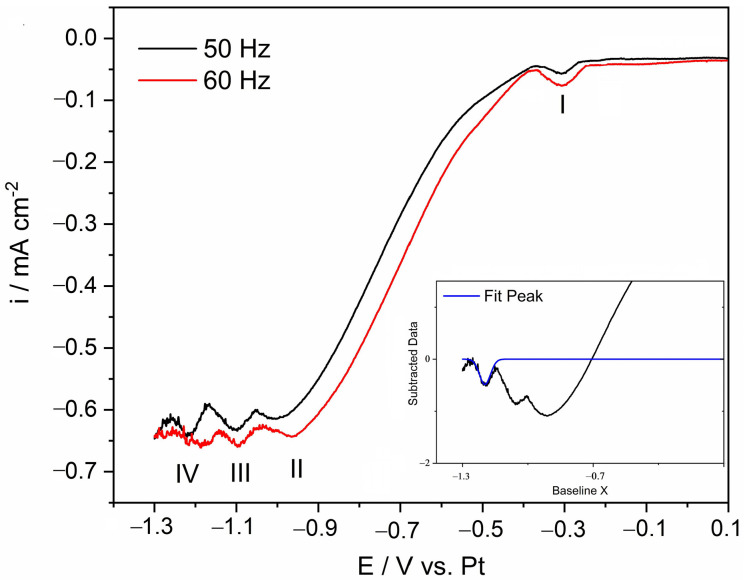
SWV obtained with different frequencies on Au WE; pulse height: 25 mV; potential step: 1 mV in reline + regolith electrolyte; temperature 343 K; insert: Gaussian fitting for 50 Hz.

The electrochemical reduction of the metallic ions in the electrolyte was further analyzed by SWV, to better understand the complex reduction mechanism involving multistep electrochemical reactions. This technique has been used to provide more clearly defined signals related to the redox transitions of cations present in the supernatant layer of the electrolyte. The electrochemical processes were examined at a step potential of 1 mV, and at different frequencies on a Au cathode in the DES electrolyte containing dissolved regolith at 343 K, as shown in Figure 2. Electrode processes associated with the redox reactions of metallic cations in the electrolyte yielded four well-defined peaks. For such fully irreversible cathodic processes involving multiple redox steps, as in this study, the number of electrons involved in a particular redox reaction can be calculated using the following equation at 298.15 K:

ΔE_p/2_ = (63.5 ± 0.5)/αn (1)

where square wave half-peak width *ΔE_p/2_* (mV) depends on the *n*, which is the number of exchanged electrons, and *α* is the transfer coefficient, commonly assumed to be 0.5 [33,34]. Since all measurements in the present study were performed at 343.15 K, Equation (1) can be expressed as:

ΔE_p/2_ = (73.1 ± 0.5)/αn. (2)

where *ΔE_p/2_* depends on the *n* [33,34]. In the present study, the applied parameters did not enable a definitive determination of the number of electrons involved in the reduction processes corresponding to cathodic peaks I–III. However, based on the aforementioned values of equilibrium potentials of metals in reline, the first cathodic peak (I) observed on SWV at −0.300 V vs. Pt can thus be associated with a reduction step of either Fe^x+^ or Cr^3+^. For FeO_T_, the combined contribution of various iron oxides in regolith implies that iron species in the electrolyte exist in different oxidation states. The onset potential for the electrochemical deposition of metallic chromium ions is assumed to follow the reduction processes of Fe^x+^. Consequently, if it is assumed that the cations in the electrolyte undergo multistep redox transitions, the individual reduction steps occur simultaneously, which hinders the designation of the precise potential range for these processes [35,36].

Cathodic peaks (II) and (III) appearing at approximately −1.040 V vs. Pt, and −1.180 V vs. Pt, respectively, probably correspond to the next steps of subsequent Fe ion and Cr ion reduction [20]. Given that the equilibrium potential of E_Si_ is more negative than those of E_Fe_ and E_Cr_ in reline, it can be concluded that cathodic peaks (II) and (III) also correspond to the reduction of Si^4+^ ions and/or bulk deposition of Si. The electrochemical reduction of Si(IV) ions in ILs proceeds through a multistep pathway, involving successive transitions from Si(IV) to Si(III), then Si(II), and finally Si(0) according to [37,38]. The cathodic peak (IV) observed on SWV at around −1.230 V vs. Pt, after Gaussian fitting (for 50 Hz *ΔE_p/_*_2_ = 0.04967 V, and for 60 Hz *ΔE_p/2_* = 0.05722 V), corresponds to an exchanged electron number on average 2.75, which can be approximated as three electrons. Conversely, assuming a three-electron transfer process (*n* = 3), Equation (2) can also be employed to determine the charge transfer coefficient (*α*) [33,34]. The experimentally determined half-peak width yielded an *αn* value of 1.47, corresponding to a charge transfer coefficient of *α* = 0.49. This value is in good agreement with the commonly assumed value of *α* ≈ 0.5 for irreversible electrode processes, indicating that the experimental results are consistent with the proposed irreversible redox process. Considering this value, together with the measured equilibrium potential of E_Al,_ which is more negative than the equilibrium potential of E_Fe_, E_Cr_ and E_Si_ in this electrolyte under the applied conditions, it can be assumed that this peak represents the one-step process of aluminum electroreduction.

### 3.3. Potentiostatic Electrolysis

To validate the hypothesis regarding the proposed sequence of metal electrodeposition based on the previously presented electrochemical reduction mechanism, electrolysis experiments were conducted in the supernatant layer of the reline + regolith electrolyte at a cathodic potential of −0.800 V vs. Pt for 4 h. The selected potential is within the range of cathodic peak (II) observed in the CV, which was previously associated, among other processes, with the reduction and deposition of Si metal. The experimental conditions were chosen to electrodeposit Si metal on the cathode.

**Figure 3:**
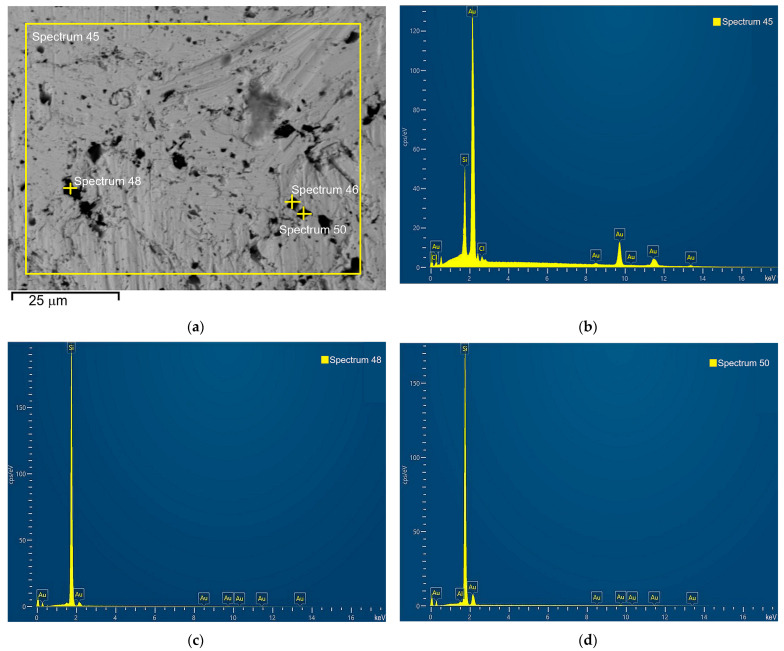
(**a**) SEM image; (**b**–**d**) EDS spectra of the Au cathode obtained after electrolysis for 4 h at cathodic potential of −0.800 V vs. Pt, from the reline + regolith electrolyte; temperature 343 K.

The resulting morphology from the deposit obtained at cathodic potential of –0.800 V vs. Pt, as shown in Figure 3a, revealed a relatively compact surface layer, although numerous dark areas with black clusters are observed. Integrated with EDS analysis, marked in yellow color on Figure 3a, metal spectra for this deposit are shown in Figure 3b–d and the results of EDS analysis are summarized in Table 2.

The results of the EDS analysis (Table 2) clearly demonstrate the presence of Si, and the content of Si reaches up to 89.6 wt%, (spectrum 48) within the dark regions on the electrode surface, as shown in Figure 3c. This indicates that the deposit, especially the dark regions on the WE, is almost entirely composed of Si without detectable impurities such as co-deposited Cr or Fe. The main deposit product was metallic Si (Figure 3c,d), confirming that the applied methodology enables selective Si electrodeposition by adjusting the electrode potential. EDS analysis indicates that Al in the deposit could be expected (Figure 3d, spectrum 50; however, its concentration was below the EDS detection limit), and Al was not detected by ICP-OES analysis. Despite the lowest values, ICP-OES analysis, apart from Si confirmed the presence of Fe and Cr in the deposit. The content of the electrodeposited Cr and Fe was approximately 0.3 wt% for Cr and 0.1 wt% for Fe, which explains the absence of characteristic signals for Fe and Cr in EDS analysis. Further investigation within the proposed approach will be crucial to demonstrate the feasibility of metal separation and recovery from regolith.

**Figure 4:**
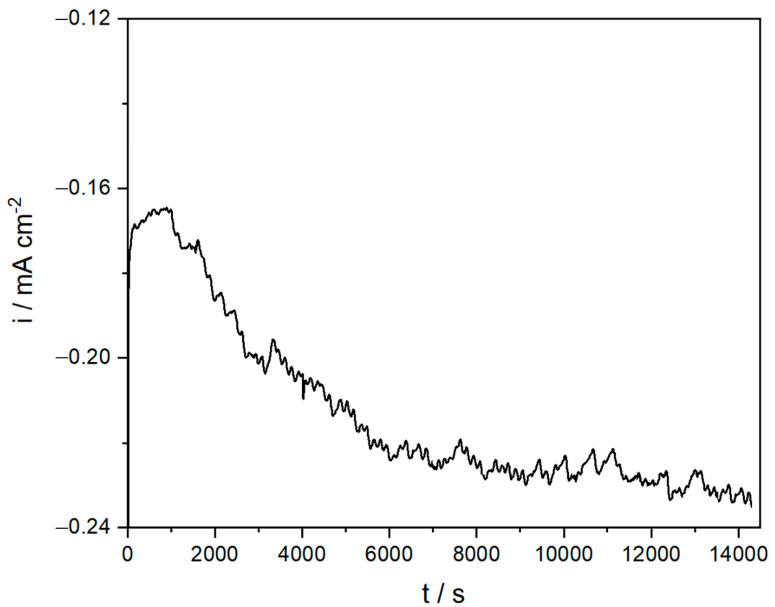
Current density (*i*) as a function (f(*t*)) during potentiostatic deposition on Au WE from the reline + regolith electrolyte at −1.250 V vs. Pt, for 4 h; temperature 343 K.

**Figure 5:**
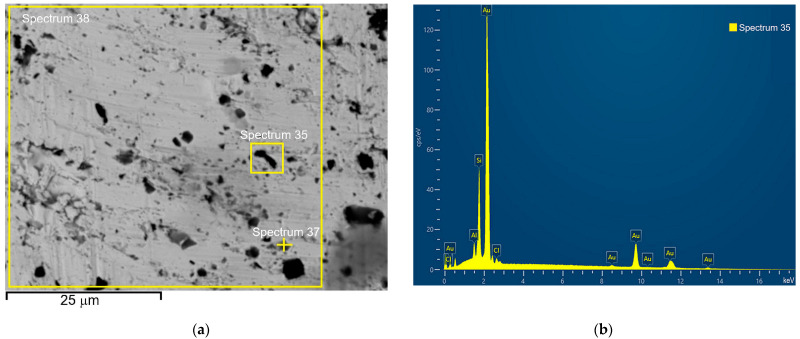

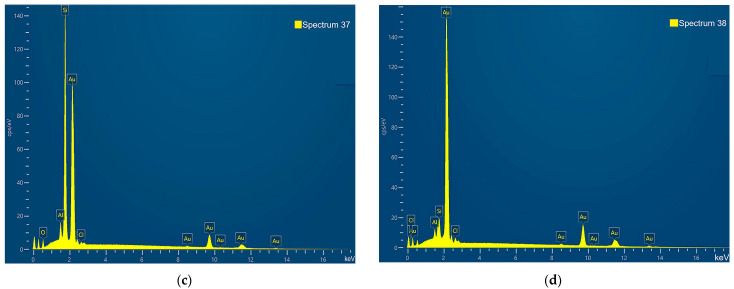
(**a**) SEM images; (**b**–**d**) EDS spectra of Au cathode after electrolysis at cathodic potential of −1.250 V vs. Pt for 4 h, from the reline + regolith electrolyte; temperature 343 K.

At a cathode potential of −1.250 V vs. Pt, which is more negative than the cathodic peak (III) observed on CV, electrolysis was performed to initiate metal deposition. Based on results from equilibrium potentials and SWV measurements, these potentials should be well within the reduction region of Al, but not negative enough to induce magnesium codeposition. Figure 4 shows the chronoamperometric response *i* = f(*t*) during potentiostatic electrolysis at a cathodic potential of −1.250 V vs. Pt, SEM and EDS analyses of the deposit are presented in Figure 5, and the results of the EDS analysis are summarized in Table 3.

The current density showed a relatively steady increase and, after gradually rising during the first two hours of electrolysis, reached approximately −0.230 mA cm^−2^. After this period, the current density stabilized within a constant range of around −0.240 mA cm^−2^, as shown in Figure 4. The relatively constant range of current density stands out as an indication that prolonged electrolysis is also feasible in the system.

As seen in Figure 5a, the resulting deposit formed a relatively compact layer on the WE surface, containing numerous black clusters. The EDS analysis in Figure 5b–d of the deposit marked in yellow on the SEM images, spectrum 35 or 37, illustrates that the Si content in the deposit reaches up to 23 wt% within the darkest area on the electrode surface. EDS analysis clearly showed that Al is electrodeposited with Al up to 1.9 wt% on the electrode surface (Table 3). There is a trace of chlorine, likely originating from the electrolyte residue, and small content of the O. This indicates that at higher overpotentials apart from Si, Al recovery is also feasible from the regolith.

**Figure 6:**
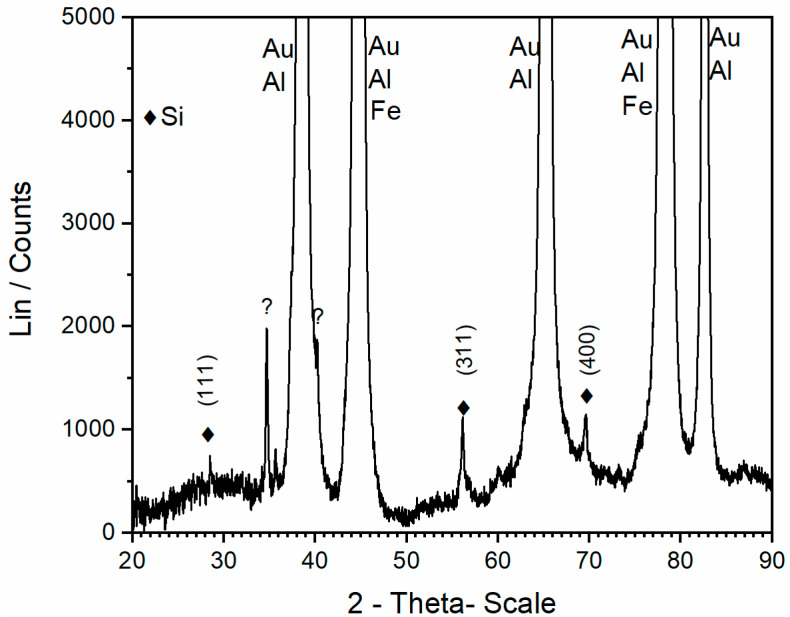
XRD analysis of the sample after 4 h of deposition at −1.250 V vs. Pt in a reline electrolyte containing dissolved regolith; WE: Au; temperature: 343 K.

After confirming the potential controlled electrolysis as a suitable mode for selective metal electrodeposition, the cathodic product was also analyzed by XRD. As shown in Figure 6, the characteristic diffraction peaks in the XRD diffractogram are from the substrate, Au [PDF# 03-065-2870]. The positions of the primary diffraction peaks characteristic of Al metal [PDF# 00-004-0787], Fe [PDF# 01-088-2324] and even Cr metal [PDF# 01-085-1336] align at almost the same 2*θ* values as those for Au. Therefore, it is difficult to unambiguously assign these signals to a specific metallic phase, such as electrodeposited Al or Fe, using only XRD. Nevertheless, several distinct diffraction peaks that could not be attributed to the substrate were observed at 2*θ* = 28.47° (111), 56.1° (311) and 69.12° (400), and correspond perfectly to the cubic crystal structure of Si [PDF# 00-27-1402].

Additional characteristic diffraction peaks corresponding to other metals were not observed in the diffractogram.

After the deposition process, the resulting deposit is subjected to ICP-OES analysis. Assuming that the analyte volume remained constant (16 mL) during both measurements, the metal removal efficiency from the electrolyte was calculated using the following equation:

(3) $$\mathrm{Metal}\;\mathrm{recovery}\;(\%)\;=\;\frac{M_{d}}{M_{0}}\;\times \;100$$

where *M_d_* is the mass (μg) of metal found in the deposit, and *M_0_* is the initial mass (μg) of metal in the electrolyte before electrodeposition. According to the ICP-OES analysis data of the resulting deposit and using Equation (3), it can be concluded that on average 7% of Si, 20% of Al, 23% of Cr and 5% of Fe were electrodeposited from the resulting electrolyte. The presence of Al in the resulting deposit when applying more negative deposition overpotentials −1.250 V vs. Pt, compared to −0.800 vs. Pt, is consistent with the reported experimental results. CV and SWV measurements alone were not sufficient to unambiguously determine the onset deposition potentials of the individual metal species present in the electrolyte. However, the combined results of potentiostatic electrodeposition at two different deposition potentials, SEM/EDS and ICP-OES, XRD characterization of the resulting deposit, together with their correlation with CV responses, SWV data, and measured equilibrium potentials, indicates that the IV reduction peak observed in the SWV corresponds to the electroreduction of Al^3+^. The experimental results demonstrate the feasibility of in situ resource utilization (ISRU) of lunar regolith using DES as the electrolyte.

## 4. Conclusions

For the first time, the metal oxides composing the lunar regolith were dissolved using a choline chloride-based electrolyte. 

In contrast to ethaline, LMS-1 in reline exhibited a significantly higher dissolution rate, which can be attributed to the additional hydrogen-bond donor sites NH and C=O groups from urea and likely, the formation of metal complexes with different coordination. The equilibrium potentials of the metals were measured and correlated with the onset potentials for metal deposition, allowing prediction of the sequence of metals electrodeposited on the cathode via electrolysis from dissolved lunar regolith in reline: Fe, Cr, Si, Al, and Mg.

The study of the electrochemical reduction of metal ions derived from lunar regolith dissolution in reline indicates a highly complex electrochemical system. The reduction processes proceed through multiple steps, with several electrochemical processes occurring within a relatively small potential interval or in parallel, making identification of deposition onset potentials very difficult. A precise assignment of individual redox transitions was not possible at this stage, indicating the need for further investigation.

Based on the electrochemical measurements, parameters for electrolysis were selected, offering a novel perspective on selective metal recovery from the DES–regolith system. By adjusting the electrode potential, the main challenge of achieving controlled and selective metal deposition was successfully overcome. Si was recovered at more positive deposition overpotentials, with the deposit consisting mainly of Si, with a small amount of Fe and Cr. At more negative potentials, in addition to silicon, chromium, and iron, aluminum was also electrodeposited.

## 5. Outlook

The results of this study demonstrate that deep eutectic solvents can serve as effective low-temperature media for both the dissolution of lunar regolith simulant and the electrochemical recovery of metals. Further work should focus on enhancing recovery efficiency and selectivity through optimization of electrolyte composition, operating conditions, and electrode materials.

A key aspect that requires further investigation is the development of a suitable anodic process for oxygen evolution within the system. The co-production of oxygen represents a major advantage for ISRU applications, as it is critical for life support and propulsion. Coupling cathodic metal recovery with anodic oxygen evolution could therefore enable an integrated electrochemical process with improved resource efficiency and potential for closed-loop operation in extraterrestrial environments.

The relatively low operating temperature of the proposed system offers clear benefits in reducing thermal demands and simplifying system design compared to conventional high-temperature molten salt technologies. Overall, continued development of deep eutectic solvent-based electrochemical approaches may support the advancement of efficient ISRU strategies, contributing to sustained lunar presence and future deep-space exploration.

## Figures and Tables

**Table 1:** Solubility of the individual oxides that compose lunar regolith in ethaline and reline at 343 K.

Oxides from LMS-1 Taken into Account	Weight Percent (wt%) in LMS	Percentage of Oxide Dissolution in ChCl:EG (wt%)	Percentage of Oxide Dissolution in ChCl:Urea (wt%)
SiO_2_	42.81	0.5	0.98
TiO_2_	4.62	-	-
Al_2_O_3_	14.13	0.3	0.4
Cr_2_O_3_	0.21	8.35	14.6
FeO_T_	7.87	1.63	2.27
MgO	18.89	2.89	4.1

**Table 2:** The elemental composition (in wt%) obtained by EDS analysis at different locations (spectra) of the SEM image shown in Figure 3a.

SEM Image	Spectrum	Au (wt%)	Si (wt%)	Cl (wt%)	Al (wt%)
Figure 3a	45	89.2	9.7	1.1	–
Figure 3a	48	10.4	89.6	–	–
Figure 3a	50	23.0	76.7	–	0.3

**Table 3:** The elemental composition (in wt%) obtained by EDS analysis at different locations (spectra) of the SEM image shown in Figure 5a.

SEM Image	Spectrum	Au (wt%)	Si (wt%)	Cl (wt%)	Al (wt%)	O (wt%)
Figure 5a	35	89.2	8.0	0.9	1.9	-
Figure 5a	37	69.8	22.7	0.5	1.9	5.2
Figure 5a	38	95.4	2.0	1.3	1.3	-

## Data Availability

The original contributions presented in this study are included in the article. Further inquiries can be directed to the corresponding author.
